# Foreign

**Published:** 1840-07-25

**Authors:** 


### FOREIGN.

### FERRALL'S CLINICAL LECTURE.—NO. 1.

*Mammary Abscess—Numerous Fistulæ—Circular compression—Cure—Necrosis of four-fifths of the Clavicle—Separation of the bone without injury to the limb—Fungus of the Antrum—Lymphatic contamination—No visceral taint.*

Amongst the cases discharged this week, the first to which I shall briefly direct your attention is that of a young woman in Joseph's ward, with enormous enlargement of the breast, and numerous fistulous openings—hectic fever, and great wasting of the frame. A patient in the last stage of phthisis could hardly present a more attenuated appearance than this young person, when entered for admission. The case is one of practical interest, because, although the treatment of mammary abscess generally is discussed in every systematic work, yet this particular condition of the breast, although of frequent occurrence, has not usually been made the subject of special consideration.

*Deep-seated Mammary Abscess, with numerous Fistulæ—Cure by circular compression.* (Reported by Mr. W. M'Mahon.)

Fanny Fanell, aged twenty years, admitted into Joseph's ward with the following symptoms:

The right mamma is enlarged to three or four times the size of the healthy one. It is irregular in shape, very heavy, and presents a number of fistulous openings on every side, freely discharging purulent matter. The integuments immediately surrounding each orifice are marked by a dusky purple tinge, and are depressed below the level of the surrounding skin. The whole breast is exquisitely painful to the touch, and is often the seat of distressing uneasiness and sense of weight.

She is greatly emaciated, very pale, and complains of excessive weakness. Bowels constipated; tongue coated slightly, but moist; pulse one hundred and ten, small, and feeble. On waking from sleep, she finds herself bathed in perspiration. No cough; no evidence of pulmonary disease.

I may here interrupt the narrative, to remind you of my observation at the time, that here was a case for the treatment of which no rule of practice was established to which I could invariably subscribe. If you consult the systematic works of character, you will be disappointed if you expect to find this case provided for. Mr. Hey is the only author who seems to direct attention to this particular state, but I believe you will meet few ladies who will submit to the operation he describes. In hospital practice, where the patient has great confidence in your humanity, she will submit with extraordinary patience to the process; and under those circumstances I have performed with success, although the hewing up of a diseased breast in all directions is any thing but an object to view with complacency. I could not help feeling, besides, that erysipelas was very likely to succeed to the infliction of numerous wounds, especially in a constitution already reduced below the standard of health. After describing accurately the condition of the mamma which succeeds to deep-seated abscess with various openings, Mr. Hey says, the cure cannot be accomplished unless the course of the sinuses be traced, and each laid open completely, however numerous they may be, or tortuous in their windings through the organ. He insists on this operation as indispensable, and even admits that, by doing it as it should be done, the breast may be "divided into several pieces." The extent to which the organ was incised may be inferred from his saying that, when any portion of the mamma was thus insulated, and rendered pendulous, his remedy was, to "remove it altogether." This was bold practice, you will say; but, from the character, experience, and singular ability of Mr. Hey, you may be satisfied that it was not lightly undertaken or recommended. If you look into other authors, as Pearson, James, Boyer, Sabatier, &c., you will find that they consider the question of opening the abscess or not, but do not seem to contemplate the degeneration of the openings into intractable fistulæ, wearing out the constitution of the individual. M. Dugès, in his elaborate and excellent memoir in the *Dictionnaire*, is equally unsatisfactory on this point. He discusses all varieties of mastoitis with great precision, but not this peculiar condition which so often succeeds to the abscess if deep-seated. Sir Astley Cooper's valuable advice on the treatment of sinuous ulcers is more in point. He recommends (without particular reference to the mammary fistulæ,) injections and pressure. The material of the injection which he seems to prefer is either port wine or tincture of lytta. It is obvious that we could not hope to make any injection follow the tortuous course of so many fistulæ passing in all and even opposite directions through the substance of the breast. Pressure applied in the ordinary way, or antero-posterior pressure, although of great service in sinuous ulcers of less extent and number, had invariably disappointed me when tried in analogous cases to the present. The pressure, however dexterously applied, had the effect of forcing together the parietes of the fistulous canals at some point of their course anterior to their extreme or further end, and thus prevent the exit of the discharge which was secreted by this distant portion of the pyogenic membrane. The consequence was, that a new depôt was formed, and a fresh attack of feverishness was set up, until it made its way in some direction or other to the surface.

It occurred to me, however, that pressure could be made available, if, by some modification in the mode of applying it, we could imitate the circular compression which so often succeeds when employed on the limbs; and the remarkable prominence of the mamma seemed to favour our design. With this view we proceeded as detailed in the next day's report.

*Jan. 16th.*—A probe is readily passed to a considerable depth on some of the fistulæ by accommodating the curve of the instrument to the direction of the canals. The orifices are covered with simple dressing or lint. A series of compresses, made of fine tow, are arranged so as to make pressure on the circumference of the breast or round its base; the anterior portion is left free for the discharge. A double-headed roller is made to pass over the compresses from beneath; the ends are brought up and crossed over to the shoulders, thence across the back, and round again to the breast as before. The circles were repeated several times, so as to make firm pressure, and acting as a sling to support the breast at the same time. She experienced considerable comfort from the support.

The constitutional treatment consists of animal food, and a little wine. Decoction of bark, with aromatic sulphuric acid, three times daily, aperient medicine being previously given.

It would be useless to read the daily reports in this case. An improvement was evident on the second day, both in the bulk of the breast and in the quantity of the discharge, which was very much lessened.

*Jan. 21st.*—Several of the ulcers are healed; a small spot, about the size of the top of the finger, on the upper surface of the breast, is red, soft, and painful; about a drachm of healthy pus escaped on puncture; the bandage is re-applied as before.

*Jan. 26th.*—Her general health is remarkably improved. The night sweats have ceased altogether, and she begins to gain flesh. There is very little discharge from the breast, which is greatly diminished in size.

*Feb. 5th.*—She is gaining strength and colour, sleeps soundly, and has an excellent appetite. The state of the breast is satisfactory. There are only two openings unhealed; and the quantity of discharge is trifling. The pressure is most comfortable to her. The size of the breast is nearly that of the other.

*Feb. 21st.*—There has been no discharge for several days. The breast is now as small as the other, but is remarkably firm and solid. Her health and flesh are perfectly restored. Discharged.

One great recommendation possessed by this mode of treatment, compared with Mr. Hey's operation, is its entire freedom from pain or danger. The moment the circular compression was effected, the patient declared she felt comfortable, and was relieved from many of the uneasy sensations she endured before. The mamma was effectually suspended and compressed at the same time, and, the anterior surface of the part being exempted, a free exit was allowed to the discharge. As the breast diminished in size, the bandage was made to advance further forward, until its most anterior portion engaged.

The great solidity which remained after the healing of the ulcers, was the inevitable consequence of the extensive hypertrophy and thickening of the cellular tissue, which was the seat as well of the original inflammation as of the purulent deposits, which succeeded each other, until the entire organ was occupied.

.The swelling and prominence of the breast was a favourable circumstance in the present case; but I should think the mode of employing pressure, adopted here, could be used in any case requiring it; for the part could be drawn gently forwards by an assistant while the compresses were being adjusted, so as to act only on the circumference or base of the organ. This would seem to be essential to its success; for it is plain, that any pressure made in front would only have the effect of opposing the free discharge from the parts behind.

The next case, amongst those discharged, is one which shows how little constitutional disturbance may be occasioned by the death and exfoliation of a large portion of a bone, possessing such important anatomical relations as the clavicle.

*Necrosis of the clavicle—Exfoliation of four-fifths of the bone at its acromial end, without deformity.* (Reported by Mr. Howel Scriven.)

Margaret Day, ætat. fifteen, was admitted into Joseph's ward with the following symptoms: an oblong ulcer occupied the centre and acromial extremity of the line of the clavicle; about an inch of this end of the bone projected at an angle through this breach, and the remainder of the ulcer presented an unhealthy surface of large pale granulations.

The projecting bone exhibits three or four irregular openings, through which a probe passes into the substance of the clavicle, and, moving freely about, shows the extent to which the cancellous structure has been removed by absorption.

There is very little pain in the part, except when it is examined, or accidentally hurt. The girl's health is good, and the motions of the shoulder are scarcely impeded. There is very little tendency in the scapula to fall forwards, although deprived of its support.

The limb is supported by the clavicle apparatus, which keeps the shoulders from inclining forwards, and the ulcer is dressed with lint.

The history of this case is briefly this:—Two months ago, after a slight hurt in the part, she felt pain, and in a few days swelling and redness appeared. She found great difficulty in using the arm, but was not prevented from going about as usual. The pain increased, and at length a discharge took place; the opening became larger; and the bone was observed to make its appearance three weeks before admission. She thinks it is coming more forward every day.

From this period to that of leaving the hospital, (four weeks,) the progress was gradual, and accompanied by no remarkable event. The bone was moved occasionally by the hand, in order to favour its separation. The shoulder was supported by the apparatus. Her general health scarcely required attention.

At length the bone was found lying detached under the dressing, and was examined carefully. The sequestrum was four inches and a half in length. It included the acromial articulating extremity entire, denuded of its cartilage, but otherwise unaltered. The articulating surface was as perfect as that of any dried clavicle from which the cartilage merely had been removed. Internal to this point, or nearer the mesial line, the openings before mentioned were observed. Farther on, the bone was smooth, hard, and compact, externally. At the inner extremity of the fragment an irregular worm-eaten appearance was remarked, and the cancellous structure was here completely removed, leaving merely the shell of the bone.

You will remember, gentlemen, that I explained to you, in the first instance, the grounds on which we were justified in leaving the separation of this necrosed bone to the efforts of nature. In the first place, no constitutional disturbance existed; and as the girl had no pressing necessity for severe exertion, the difference of the time so occupied, compared with that which an operation and subsequent healing would have required, was of little importance. In the next place, there was no evidence that any new shell had been formed, in which case the important parts, beneath or behind the clavicle, might be injured or disturbed by any hasty attempt to remove it. The bone was firmly adherent at its inner end on her admission; and you perceive, by examining this specimen, that no traces of absorption are visible in the middle portion of the fragment.

The absence of a new shell of bone, which, in ordinary cases, and in other bones, incloses the sequestrum, will, in this case, account for the early separation of the injured portion. If a new formation had taken place, a solid obstacle would have been opposed to the removal of the fragment. It is curious, however, to remark, that although no substitute had been prepared to do the office of the clavicle, yet the motions and even the power of the arm were little, if at all impaired. When we recollect that this bone is the point of attachment for many muscles concerned in the movements of the arm and head, we must be struck with the powers of accommodation possessed by living structures, when not too suddenly injured. The clavicle is broken by a fall, and at once the arm droops helpless, and the point of the shoulder falls forward on the chest. But four-fifths of the bone may be altogether removed by a slower process, and yet neither of these accidents will occur to any remarkable extent. It has even happened that the entire clavicle has exfoliated, or been thrown off by necrosis, without any loss of power of the limb. A case of this kind is recorded in the Memoirs'of the Academy of Surgery, Paris, a work replete with valuable facts. In the instance there related, a new clavicle was subsequently formed, and the articulations with the sternum and acromion were affected as in the original bone. In our patient, although the part is healed, there is no evidence of bony deposit as yet. The cicatrix has a firm but yielding feel, and the part can be pushed back without any sense of resistance being communicated greater than belongs to a fibrinous or cartilaginous structure. There can be little doubt, however, that, in due time, new bone will be formed to unite with the portion of the old clavicle which remains attached to the sternum, and to articulate with the acromion at its other extremity. The apparatus is still worn, as calculated to maintain the proper distance between the sternum and shoulder, and cause the new bone to occupy the necessary extent. As to deformity, it is really surprising how little flattening is perceptible, and the cicatrix is becoming narrower every day. I have seen the cicatrix of scrofulous ulceration much more remarkable in every way; and there is reason to hope that when new bone is formed behind it, there will be little to attract attention.

The long-expected death of Ford, the poor woman with fungus of the antrum, affords us an opportunity of ascertaining the condition of the principal organs, and of testing the opinion I entertained as to the nature of the lymphatic swelling which existed beneath the jaw. I shall first read a few of the notes of her case, to refresh your recollection of the appearance of the parts during life.

*Fungus of the antrum—Lymphatic contamination—No visceral taint.* (Reported by Dr. Drought Dickson.)

Catharine Ford, æt. forty-five, admitted into St. Mary's ward, Nov. 5, 1838. She states that for several months before her admission she suffered from pain and swelling of the left cheek, with discharge of bloody fluid occasionally from the nostril of that side. The pain resembled toothach, and excited no apprehension in her mind. The swelling was at first attributed to the same cause, and was disregarded until it had acquired a considerable size.

The tumour occupies the entire region of the superior maxillary bone of the left side. The left eye is much more prominent than the right, and on a plane superior to it. The distance between the inner canthus and nose is much greater than on the opposite side, and the usual depression is filled up by the tumour, which here forms a distinct swelling. The canine fossa is entirely obliterated. The left ali nasa is drawn downwards, and considerably elongated. A fungoid mass is beginning to appear in this nostril, and exudes a fœtid discharge of a brownish colour and sero-purulent appearance. Within the mouth the palate of the left side, and extending even beyond the middle line, is occupied by an irregular bulging mass, covered only by the mucous membrane. The bone has been absorbed, and the swelling affords only the resistance of elasticity. She is much emaciated, and sallow in colour. Her rest is disturbed by the discharge from the nose flowing back into the fauces.

Beneath the jaw-bone, in the digastric space, a lymphatic, enlarged to the size of an almond, is to be felt; it is remarkably hard to the touch, but is moveable: she has no cough.

These were the notes made at the date of her admission. The extent of parts involved, the extensive destruction of the bones in all directions around the morbid growth, together with the exhausted state of the poor woman, seemed to forbid any operative project. But when we considered that a lymphatic gland had already undergone a morbid change, and that its consistence rendered it very probable, that this change was that of schirroma, I gave up all hope from such a proceeding.

The principal changes that occurred within the first month of her residence in the hospital, were, the further protrusion and displacement of the eye, and the exposure of the fungus in the mouth, by the giving way of the mucous membrane of the palate. The globe of the eye was displaced more and more every week, and gave to the face that peculiarly hideous expression which is observable in these deplorable cases. At length the axis of the eye was altogether directed outwards and upwards, and the organ protruded almost entirely beyond the eyelids. Notwithstanding all this, vision remained perfect, and she could recognise every person around her, when the opposite eye was closed. The double source of fœtid discharge which oozed continually now into the nostrils and mouth, became at length her principal source of complaint, for she had for some time suffered very little from the original pain. It appeared, that the pain diminished in proportion to the destruction of the bones and the free expansion of the tumour.

Early in January she complained of pain in the cheek about its middle. The integuments were inflamed, and a circumscribed swelling distinct from that within was formed. A poultice was applied, and after seven or eight days distinct fluctuation was perceptible in its centre. I made a puncture here, and gave exit to about three drachms of healthy pus. A probe passed into the sac of the abscess gave no indication of its communicating with the antrum. It appeared to be in the substance of the cheek itself. I called your attention to this circumstance at the time, for I deemed it worthy of remark, that a healthy abscess should form over a mass of morbid growth, merely as it would seem from the irritation occasioned by its presence at some distance from the cellular tissue in which pus was formed. I reminded you that analogous collections of matter are occasionally observed in the cellular tissue external to a carious joint, without any communication with disease within. This superficial abscess in Ford's case continued to discharge healthy matter for a few days, and then healed.

From this period until that of her death, in the latter end of January, a degree of somnolency was added to her other symptoms. Her utterance was imperfect, and the constant discharge of fœtid ichor, a good deal of which passed into her stomach, destroyed all relish for food: she could not be prevailed on to take nourishment, and her emaciation rapidly increased. She died, in fact, like those who sink from organic disease of the stomach, as much from inanition as from the irritation occasioned by the disease.

*Necropsy, twelve hours, post mortem.*—You had this morning an opportunity of witnessing the result as follows:

Chest.—The lungs were sound; a few points of adhesion to the costal pleura were observed towards the base of the right lung posteriorly. The heart was small: the walls bore a reasonable proportion to its size in their thickness. There was no valvular disease.

Abdomen.—The liver was of moderate size; its white tissues rather prevailed, but not to any remarkable degree; carefully made sections in different directions failed to discover any deposit or organic change.

The spleen was of the ordinary hue. The stomach distended with air, and containing some remains of her drinks, with a quantity of glairy mucus. The colour of the mucous membrane was perhaps a little darker than usual, but no vascularity of an arborescent character was observed. There was nothing worthy of remark in the intestines, kidneys, or other parts. The diseased mass was removed, and you here observe the extensive destruction of parts which it occasioned. The osseous boundaries of the antrum are almost completely removed by absorption. The displacement and disorganization of bones and soft parts in the vicinity of the tumour are remarkable. The structure of the fungus is unequal. You see that it is partly fibrous, and that other portions exhibit a homogeneous and almost lardaceous appearance on section. There is no appearance of softening in any part. We shall now proceed to examine the lymphatic gland, which acquired so little additional bulk while under our observation.

You perceive it is remarkably dense, and cuts like fibro-cartilage; it consists of striæ of transparent pearly colour, between which a yellow texture is to be seen. The latter does not seem to be pulpy, but is evidently less consistent than the other.

So far, then, our diagnosis of the structure of this gland is justified. It is decidedly of scirrhous character; and although remaining for months with very little increase of growth, I cannot help thinking that if other circumstances had encouraged the performance of an operation, or afforded the least chance of its success, this lymphatic gland, so degenerated, and having taken on the characters of schirroma, would have been excited into activity, and destroyed the poor woman as effectually as the original disease. Considering the extent of the growth at the period of her presenting herself, and that the mouth, nose, and orbit, were involved in the disease, no surgeon of ordinary prudence and humanity could entertain the idea of an operation.

The post-mortem examination, however, concurs with my own previous experience, and that of others with whom I have conversed, in supporting the probability in such cases of the absence of visceral taint. You are aware that in fungoid growths on other situations, the chances of success from operation are greatly lessened by the frequency of similar morbid deposits in the liver or other organs.

Our means of making minute examination or anatomical analysis of such growths, are not yet sufficiently perfect to enable us to decide whether there be any essential difference between the fungus of the antrum of a malignant appearance, and those which are found elsewhere. But the case before us is quite different from the milder tumours which occupy this situation. Even in its earlier stages it threw out a fungoid mass into the nose, bleeding occasionally, exuding a fœtid sanies, and accompanied with a visible decline of health. I would not, however, class it with the medullary fungus, although it seems entitled to some place intermediate between it and the fibrous tumour of the antrum. The peculiar structure which you observe in the lymphatic gland would favour this opinion, and certainly suggest the rule that when such a tumour is discovered in the course of the absorbents, great caution should be used in deciding on interference by operation.—*Lon. Med. Gaz.*

---

### ROYAL MEDICO-CHIRURGICAL SOCIETY.

May 12th, 1840.

*On Aneurisms, and especially Spontaneous Varicose Aneurisms of the Ascending Aorta and Sinuses of Valsalva, with Cases.* By John Thurnam.—After some observations on the probable course and termination of aneurisms limited to the lesser aortic sinuses of Valsalva, the author proceeded to the proper subject of his paper, on *spontaneous* varicose aneurism of the aorta, a form of disease which is new to pathologists. This lesion he stated to have been entirely overlooked by M. Breschet, in his valuable memoir on varicose aneurism, although Mr. Syme had already detailed a case seated in the abdominal aorta and cava. After adverting to the interesting case published by Mr. Perry, in the 20th volume of the Transactions of this Society, the author proceeded to the consideration of the lesion as occurring in the ascending aorta. He considered this part of the arterial system, including the aortic sinuses, as more liable than any other to the formation of such spontaneous intervascular communications, in consequence, principally, of its close contact with various parts of the venous system. He detailed eleven cases, and referred to the preparations from six others, in which spontaneous varicose aneurism had existed. Of these, two were seated in the descending aorta and inferior vena cava, and one in the arteria innominata and superior vena cava. The others were all seated in the ascending aorta (excepting one in the arch,) and communicated, one with the superior vena cava, two with the right auricle, one with the right ventricle, and ten with the pulmonary artery.

He then proceeded to give the history of the disease, which he founded upon an analysis and comparison of these cases. He stated that the mode of communication between the aneurismal sacs and the venous system might occur in two principal ways, viz., either suddenly, and by rupture, in consequence of some effort on the part of the patient, or in a more slow and insidious way, by softening or ulceration of the walls of the sac. The symptoms which announce the formation of the varicose aneurism, under the first of these circumstances, were described, and were stated to resemble those of a rupture of the heart. The symptoms of the disease were divided into those connected, firstly, with the external surface and system generally; secondly, with the respiration; and thirdly, with the state of the heart and great vessels. The most important of the general diagnostic signs were stated to be, livor of the surface, or a distended and even varicose condition of the subcutaneous and other veins; severe and rapidly advancing anasarca; all these symptoms being limited to such portions of the body as are below, or the venous system of which is distal to the varicose orifice. When the varicose aneurism is between the descending aorta and inferior cava, the legs, scrotum, and lower half of the body, when between the ascending aorta and the superior cava, the arms, face, and upper half of the body, and when between the ascending aorta and one of the right cavities of the heart, or the pulmonary artery, the whole of the body is the seat of the dropsical effusion. The dyspnœa is usually severe, and often attended by cough and bloody expectoration. The pulse is *remarkably jerking*, and there are, frequently, great emaciation, debility, loss of muscular power, and deficient animal heat, with sensorial disturbance, in the shape of delirium or coma. The physical signs are stated to be "a superficial, harsh, and peculiarly intense sawing or blowing sound, accompanied by an equally marked purring tremor, heard over the varicose orifice, and in the current of the circulation beyond it: this sound is continuous, but is loudest during the systole, less loud during the diastole, and still less so during the interval. The characters of the sound, as regards intensity and continuousness, will probably altogether distinguish it from any that is heard in ordinary cases of aneurism, or in valvular diseases of the heart."

The author then entered upon the consideration of the pathology, prognosis, and treatment of the lesion, including the rationale of the physical signs.

He also drew an interesting parallel between the symptoms of internal spontaneous varicose aneurisms, as developed in his paper, and those of the ordinary external or traumatic varicose aneurisms, as described by Hunter, Cleghorn, Scarpa, and Breschet.

The paper was concluded by some observations on aneurisms of the ascending aorta rupturing into the left cavities of the heart, and two illustrative cases were narrated.

—

### PATHOLOGICAL DEPARTMENT.

May 19, 1840.

*Hydatids of the Uterus.*—Mr. North exhibited a large quantity of hydatids recently expelled from the uterus of a patient under his care. They consisted of a numerous collection of small-sized cysts, like small grapes or currants, attached together by pedicles, or floating loosely in a bloody-coloured fluid. The lady was presumed to be between the fourth and fifth month of her pregnancy. Previous to their expulsion she had slight attacks of hæmorrhage, and occasional discharges of a watery fluid from the uterus.

After making some remarks as to the unfitness of the term *hydatid*, usually applied to these bodies to designate their true character, Mr. North proceeded to observe, that, by all the best pathologists of the present day, it was agreed that they were a form of blighted or diseased ovum; that they were the result of a morbid change in the chorion. It appeared that these uviform cysts, clustered together like currants, were an enlarged or exaggerated condition of the small cysts attached by pedicles, which form the principal structure of the fleecy chorion in the natural ovum. That such opinion is well founded, seemed to be proved by a series of about six preparations, which he had brought for the inspection of the members. These were aborted ova, in different stages of disease resembling that of the specimen on the table. In some of these preparations the chorion retained, in certain places, very nearly what we should allow was the natural structure of that membrane; while, in different parts of the same specimens, distinct appearances of the disease in question were manifest. A gradual conversion of the membrane from its normal condition to the diseased state presented in the specimen which he had just obtained, could be observed in the series of preparations.

Having offered some observations, in the next place, on the opinions commonly entertained as to the origin of this disease of the ovum, and as to the symptoms indicating its occurrence, as well as to the prognosis, he proposed the question to the Society—Whether the same kind of cysts or hydatids were ever expelled from the virgin uterus; in other words, whether this disease invariably depended or not on the presence of an ovum in the uterus? He knew that authorities of the highest name had declared that no authentic cases were on record, of a similar set of bodies being discharged by females, in whom there might not be well-founded suspicions of their having been pregnant. He himself, however, was acquainted with two cases, originally published in a Glasgow journal, by Dr. Andrews, where, from the facts detailed, he had no doubt that the cysts which were expelled were exactly of the same nature as those in the preparations on the table; and the females—young unmarried girls, in a respectable sphere of life—were altogether free from the suspicion of their having become pregnant. He put it to the Society whether there was any morbid condition of the uterus, independent of impregnation, by which the discharge of such hydatids could be satisfactorily accounted for. He was aware, and it was a point of importance to remember, that if, after delivery, a portion of the placenta remained adherent to the uterus, a change might take place in the structure of that fragment; so that, in process of time, a disease of the nature at present under discussion might be generated from it; and accordingly, a female—for example, a widow—might have a discharge of such hydatids from the womb, without any fresh impregnation. But in a medico-legal point of view, as well as for other obvious reasons, it was important to determine whether the hydatids referred to were ever found in a uterus where conception had not taken place; and that question he begged to put to the members of the Society.

Mr. E. Wilson was of opinion that the particular disease under discussion was invariably the production of an impregnated ovum morbidly affected. He stated his belief, although he was not prepared to give proofs of his views, that, in virgins, ova frequently escaped from the vesicles of De Graaff, and were conveyed along the Fallopian tubes to the interior of the uterus. But such ova were never matured—never had a chorion investing them. The formation of this membrane could only occur as a consequence of impregnation; and as the production of the so called hydatids depended, in his opinion, on the presence of the chorion, he regarded the existence of the disease as certain evidence of previous conception.

Dr. Hodgkin coincided with the last speaker in considering that the cluster of cysts, very erroneously and absurdly regarded by some as entozoa, and hence termed hydatids, were always to be traced to the previous existence of an impregnated ovum within the uterus. The fœtus was seldom or never to be detected. Hence, if we pleased, in order to relieve the minds of friends, when doubts gave rise to uneasiness, we might say that this evidence was wanting to establish the fact of conception: and yet how the fœtus, in such a morbid condition of the chorion, should not undergo its proper development, and should not be recognizable in the discharged mass, we cannot be at a difficulty to understand. He had frequently and for many years back, examined the appearance of the cysts in question with particular care, and he was convinced that they were degenerated vessels of the chorion, a peculiar form of dilated vessels, the trunks and ramifications of which were more or less visible in different stages of the disease; and served to connect the cysts together in their characteristic clustered form. He did not altogether agree with Mr. E. Wilson, in supposing that ova could be transmitted, in the virgin, from the ovaries to the cavity of the uterus, or that they were conveyed along the Fallopian tubes. He did not doubt that the vesicles of De Graaff were frequently ruptured in virgin females; but as to what took place in such occurrences afterwards, he did not venture, from want of evidence, to express any opinion.

*Two Cases of Tumours situated between the Cerebellum and Pons Varolii, and compressing theseparts.*—Mr. Shaw exhibited two specimens obtained from different patients, of tumours lodged in corresponding situations at the base of the skull, and in neither case had any distinct cerebral symptoms been manifested during life.

The first specimen was removed from a female, 69 years of age, brought into the dissecting room of the Middlesex Hospital School, during the past session. The required certificate of the medical attendant attested that she had died of asthma. On turning out the brain for the purpose of dissection, a tumour somewhat larger than a pigeon's egg was found lodged between the right lobe of the cerebellum and the inferior part of the pons varolii. One third of the tumour was imbedded in the part corresponding in situation with the crus cerebelli, while the remaining two thirds projected above the level of the cerebellum. A considerable indentation was formed, for the lodgment of the lateral part of the tumour, in the side of the pons varolii. A lobe of the tumour, of the size of the point of the thumb, was also found fitting into a corresponding depression, or excavation, in the petrous portion of the temporal bone. The preparation of the temporal bone, containing this deep sulcus for the reception of a part of the tumour, was exhibited by Mr. Shaw, and it was seen to have been formed in the situation of the foramen auditorium internum. The margins of this foramen were absorbed to some extent around; so that the interior of the cochlea was disclosed to view, and the canal for the transmission of the portio dura was seen commencing near the hiatus Fallopii, on the anterior aspect of the bone. Notwithstanding there was this extensive removal of densely formed bone, there were no indications of disease in the immediately adjoining parts: a thin membrane, continuous with the dura mater, covered the surface of the depression, and the lobe of the tumour, although inserted into the sulcus, did not adhere to its bottom; but, on the contrary, was easily turned out from it. It was particularly observed that all the structures surrounding the tumour presented a perfectly natural appearance; that is, no adhesions were contracted; there was no opacity of the membranes, and, on the whole, there were no indications of the presence of the tumour having given rise to inflammatory action.

The only nerves which were directly affected in their course by the tumour, were, the portio mollis, and portio dura of the seventh pair. In regard to the former, no trace of it could be discovered; and it was inferred that it was totally destroyed. In reference to the portio dura, this nerve was seen taking its course over the surface of the tumour, and mounting upon its most prominent part. It was closely adherent to the investing membrane of the tumour, and was spread in a flattened, ribbon-like form upon it. Its fibrils retained the white, pearly lustre which characterizes the nerves at their origins.

Being curious, from finding these appearances, to ascertain more precisely the condition of the patient during life, Mr. Shaw visited the surgeon of the infirmary where she died. He learned that the woman had been long an inmate of the work-house; but that there were no symptoms which the medical gentleman could ascribe to cerebral affection. She had been bedridden for some time, but could nevertheless walk when required to get out of bed. She had no paralysis, had not been subject to fits of any kind, and her intellects were not perceptibly impaired. Particular inquiries were made as to whether the muscles of her face were paralysed; but it was stated that no defect of motion had been observed in them. This statement corresponded with the appearance of the face in the dead body, which did not exhibit any signs of distortion, or of the inflammation in the conjunctiva of the eye, so common in cases of paralysis from disease of the portio dura. The muscles, also, whose action is controlled by this nerve, were found on dissection to present the usual red fleshy appearance of muscles possessed of their natural powers, instead of having the white blanched appearance which distinguishes muscles deprived of their action for some time.

The second specimen was taken from a lady, whom Mr. Shaw had frequent opportunities of seeing during her life time. The tumour in this case was also about the size of a pigeon's egg. It was situated upon the crus cerebelli, to which it was attached by a narrow base; and it had formed a considerable indentation upon the side of the pons varolii, to which it was also slightly adherent. It contained within it a fluid of the colour of urine, the walls being composed of a substance in point of consistence between membrane and medullary matter, and possessing considerable thickness. In this patient there were no symptoms till within a few days of her death, of any general cerebral affection, such as stupor, paralysis, or convulsions. But she suffered intense and constant pain in all the surfaces of the head, and, moreover, was deprived of the sense of touch, in the same parts where the pain was experienced. The cause of this acute pain, and want of sense to impressions, is to be understood when we observe that the fifth pair was completely involved in the substance of the tumour. The disease has had the effect of insulating the sensitive surfaces of the head, supplied by the fifth nerve, from the sensorium; while, by the irritation which the tumour produced, painful sensations, referrible to the parts supplied by the nerve, were experienced at the same time.

Dr. Williams observed, that the cases just related by Mr. Shaw offered striking illustrations of the extent of change which the brain might sometimes undergo in its most important parts, when that change was effected gradually. From the appearance of the tumours there can be no doubt that they were slow in their growth, and had been unattended in their development with inflammatory action. He had, of late, enjoyed several opportunities of witnessing changes of a remarkable extent in the substance of the brain, where the symptoms of disease had been most obscure, if not totally absent. One of the cases he shortly related. It was that of a man who, eight years before his death, had suffered from fracture of the skull, with depression, but who had recovered. He was subsequently seized with paraplegia, from the complicated effects of which he died. Upon examination of the brain, there was a very considerable depression of a part of one of the hemispheres, at the seat of the fracture. It was also found that, in various distinct parts, ramollissement had taken place. Another appearance presented itself, which he had particularly noticed, but which he had not seen described in any works on pathology; that is, upon lifting up the pia mater from the surface of the convolutions of the brain, at the seat of the fracture, he found that a thin scale or layer of the cineritious matter could easily be lifted up along with the membrane; and on minutely inspecting the surface from which this layer had been detached, it presented a smooth and even appearance. To proceed with the account of the dissection: not feeling satisfied that the paralysis of the lower extremities could be the result of the disease found in the brain, especially considering that all the superior parts of the body retained their functions properly, he next examined the spinal marrow with care. It was found that this part presented a healthy appearance, except about a hand's breadth above the commencement of the cauda equina; here, upon one of the roots of the nerves, a small hard body, like a ganglion, was discovered, and in close connection with this gangliform growth, a dense body, like a fibrinous clot, and of the size of a bean, pressing upon the anterior column of the spinal cord, was found.

Mr. Shaw observed, that the remarks made by Dr. Williams were interesting, as they corresponded so exactly with observations made on a previous evening, when tumours of a large growth, compressing the substance of the brain, were exhibited before the society. He had brought, for the inspection of the members, a preparation which, he considered, showed, in a very remarkable manner, how great a change may be produced in a part of the brain, distinguished above all others for its importance in regulating the vital actions of the frame, and that part, notwithstanding the change, retaining its functions. There is no portion of the central organs of the nervous system, upon which all the actions important for preserving life so directly and immediately depend, as the medulla oblongata, or that part where the spinal marrow is united to the columns prolonged from the brain; that is, at the foramen magnum, and arches of the atlas and dentata, the two first vertebræ of the neck. Here, if we wish to destroy life instantaneously in an animal, we introduce the knife, and all motion is at once arrested; the animal dying without a struggle, or even a heave of the chest. In this preparation it may be seen what a surprising change has been produced in the relative position to each other, of the occipital bone and the superior vertebræ of the neck, which surround and protect the part of the nervous system here referred to. Anchylosis has taken place between the articulating surfaces of the occipital bone and the atlas and dentata respectively, and besides that, the bones have been displaced, previous to their union, in such a manner that the greatest degree of distortion has been the consequence. The spinal canal has consequently been completely altered in its form, so that in place of its easily admitting the points of two or three fingers at its commencement, which would have marked its natural condition, it is with difficulty that a common pencil-case can be passed through it in any part.

He could not communicate many particulars regarding the patient from whom this specimen was taken; yet those with which he was acquainted were sufficient to show, that the man had survived the occurrence of the changes alluded to for some time. The preparation was obtained some years ago, from a man who had been picked up in one of the streets in the neighborhood, in the middle of the night, in a state of insensibility, and who was brought by policemen to the Middlesex Hospital, no one being able to give an account of the accident. He died soon after his admission. Upon examining the brain, a fracture was discovered at the base of the skull, surrounding the foramen magnum, at some little distance from its margin, and presenting several angular points in the course of the fissure, some of which points had pierced the base of the brain. From the firm, consolidated appearance of the anchylosis, and the general aspect of the preserved bones, it is manifest that the disease which gave rise to the displacement must have been long completely cured; and it is to be presumed, from the circumstances in which the patient was found before his death, that he had the power of moving about without assistance.

Mr. E. Wilson stated, in reference to the observations by Dr. Williams, as to a thin layer of the cineritious substance of the brain being easily separable, in some cases, from that beneath it, that on making a post mortem examination recently, at Hanwell, of a patient who died insane, he had made a similar observation: and in drawing the attention of Dr. Conolly, the physician of the establishment, to the fact, that gentleman had stated that it was an appearance which he had seen in several patients, the subjects of insanity.

Dr. Hodgkin observed, that the appearance referred to had not escaped his notice; and he believed it had been described by authors He related some particulars of a case in which a tumour of large dimensions was found imbedded in the substance of the brain.

*Fibrinous Clot in the Arch of the Aorta.*—Dr. Clendinning, at the close of the meeting, begged to exhibit a specimen, in which a clot of blood was seen plugging the termination of the arch of the aorta. He introduced the specimen chiefly for the purpose of stating, that in the interior of the fibrinous accumulation, a fluid, possessing all the common appearances of pus, had been observed occupying a distinct cavity.

Mr. Gulliver observed, that the fluid resembling pus, referred to by Dr. Clendinning, was altogether of a different nature from pus; both chemically and microscopically this difference could be established. The fluid in question has a greater tendency than pus to submit to the putrefactive process, and does not form the same compounds with alkalies which pus so readily forms. Again, examined under the microscope, it is seen to contain numerous minute granules, not perceived in pus, and only a tenth of the size of the pus globules. When put between thin plates of glass, it does not give rise to the iridescence displayed by pus in similar circumstances. The importance of this distinction in reference to theories at present much in vogue, concerning the supposed effects of absorption of pus, or the circulation of pus in the blood, he illustrated by several interesting observations which he had recently made.

*London Medical Gazette.*

---

*On the Intermittent and Remittent Fevers of Peru.* By ARCHIBALD SMITH, M. D.—The autumnal fevers are often severe, and during the months of April and May they are exceedingly prevalent.

The type under which these fevers most frequently appear is allowed to be the tertian; and hence the natives, very indiscriminately, call intermittents, whether of quotidian or tertian form, by the vulgar name *Tercianas.*

In those parts of the Peruvian coast which approach nearer to the equator, fevers of this nature are milder than in other and more humid situations that lie to the south of Lima; but everywhere along the maritime valleys, if the case be left to nature, visceral obstructions and dropsies naturally follow protracted agues.

About the middle of April, the periodical rains nearly cease in the mountainous districts, and, consequently, the rivers on the coast begin, at the same time, to fall rapidly. Now it is that the marshes in the neighbourhood of towns and villages partially dry up, as the supply from the seasonal rains of the inland country disappears; and from these marshes and the canals for irrigation, (whose slimy channels are more and more exposed, as the volume of water flowing into them from the great streams or rivers is diminished,) miasmatic matter and a pestiferous stench are emitted.

Owing to such causes, the seaport town of Pisco used to suffer very much from *tercianas* (as the seaport of Callao still does, on account of the neglect of drainage,) till the year 1834, when the inhabitants of this town, as one of them informed me, resolved upon opening a wide drain or canal round the place. The result of this useful labour was, that the drainage became complete; and on the following year there was little or no terciana in Pisco.*

Intermittents are often observed to come on masked under a continued or remittent form; and on some occasions they are complicated with a local inflammation, as acute hepatitis for example, of which such fevers may at first appear to be only symptomatic. They do not discover their natural type or tendency for a few days; and hence the current saying among the natives,—"todavia no se ha declarado la terciana," viz. the tertian has not yet unmasked or declared itself.

In the months of April and May a great number of masked cases are observed to commence with symptoms of common catarrh, repletion, or constipation, (Empacho;) and after free alvine evacuations are procured, such affections usually declare themselves, and appear in the undisguised form of an established tertian or quotidian; or, if the fever was remittent in its commencement, it then takes on the regular intermitting type.

The general treatment of ordinary tercianas I shall notice before offering any observations on the management of more urgent cases.

In the cold fit of the milder forms of such fevers, the natives seldom do more than wrap the patient well up in bed-clothes; give some warm diluent drink; and apply warm bottles, bricks, or flannels to the feet. In the cold stage I have often given an opiate with decided advantage, and in the hot stage have had recourse to opiates conjoined with *Liq. Acet. Ammon.*, which produced the desired effect of quieting nervous irritation and promoting sweat; but these remedies are little if at all used, under similar circumstances, by native practitioners.

In the hot stage cold drink is importunately desired by the patient; and iced-water, with or without the addition of acidulating vegetable juices, is in general safe, and may be considered the most grateful and salutary beverage that can be given, except in cases of acute internal inflammation, in which, as experience teaches, such frigid draughts are inadmissible.

These iced drinks are known to quiet the feverish restlessness and oppression at the præcordia, that usually accompany the hot stage, and they soothe the general nervous excitement, together with the mental uneasiness and despondency which frequently concur to render the hot stage of an intermittent the occasion of a peculiarly distressing state of disturbed sensation and feeling. Such cooling drinks are found, as confirmed by daily and common experience, to reduce the excessive heat and excitement of the internal organs; to moderate thirst, relax the exhalents, and favour perspiration. As the excessive irritation on the internal surfaces is allayed by the sedative action of iced-drinks, this change is soon followed by the sympathetic relaxation of the cutaneous vessels, which opens the way to free and general diaphoresis, that cools the body and terminates the paroxysm. It will also be observed, during the warm summer months, that when the excitement and fever run high, perspiration is invited by light covering, while on the other hand, a load of bed-clothes would only serve to increase the restlessness and oppression, and retard the out-coming of a refreshing sweat, which prepares the patient for taking the infusion of bark, aided by some neutral salt. In some cases the bark may be dispensed with, for drinks of mallow decoction with cream of tartar, and the repeated administration of laxative enemata, are popular and frequently efficient remedies in common terciana.

After the solution of the entire paroxysm, some feel quite well and ready for a good meal, while others experience the utmost degree of lassitude, and feel little or no relish for food.

It may be observed regarding those affected in the latter manner, that, from some peculiarity of constitution, the nervous excitement which accompanies the fever is particularly intense, so that, unless active measures be early resorted to in the state of apyrexia, a few repetitions of the fever will greatly exhaust the sufferer.

* The advantage of drainage in preventing disease is well illustrated by the observation of a gentleman who had resided for several years at Arica, on the southern coast of Peru. He says:—"In former times, the water from the mountains was allowed to find its way to the sea, by running along the almost level ground in the vicinity of Arica. The consequence was the formation of a swamp, whence exhalations rose, bearing disease, misery, and death to the victims of resistless, arbitrary power. When the patriots first got possession of Arica, they cut a considerable ditch or channel on the north side of the town, to let the water have free course into the sea. The place was greatly improved, and rendered much more salubrious during the interval of time that elapsed between the period when the above-mentioned improvement was effected, and the reöccupation of Arica by the Spanish loyalists, who, with diabolical ingenuity and perseverance, filled up, or otherwise destroyed the canal, and again Arica became what it had been for centuries before, very unhealthy." See in Glasgow Medical Journal, No. xix., August, 1832, an article on the Medical Topography of some parts of Peru, relative to Dysentery and Intermittent Fever, by Mathie Hamilton, formerly Surgeon to the Potosi, &c. Mining Company.

Though the routine practice in Lima during the paroxysm of a common *terciana* be such as is here described, there occur numerous instances where fevers, essentially of this nature, do not run so regular a course, and in which, without the preliminary measure of bleeding, either before or during the exacerbation, cold drinks fail in producing a copious sweat, and a consequent termination of the fever. Of venesection during the cold fit, I have no experience.

In those severe cases where the vascular action and fever are intense, it will be commonly found that the treatment should begin with a general bleeding, to which, in most instances, the warm bath will prove an agreeable and efficacious auxiliary. Thus, when the patient is young and vigorous, the pulse strong and frequent; the skin dry and hot; and the fever attended with parched fauces, thirst, and an inward sensation of heat, or with a short and troublesome cough, which passes off with each successive paroxysm,—in such circumstances the lancet may be usefully employed. When the breathing is greatly oppressed, with the neck and countenance unusually turgid, general restlessness excessive, and the mind anxious, or the thoughts incoherent, abstraction of blood is urgently required, and cannot be omitted without hazard to the sick.

In those attacks where the fever has not unmasked itself, but has remained in a slightly remittent or continuous state for several days, and appears in company with some local inflammation, it is usually treated as if it were merely a sympathetic inflammatory fever. It is, however, remarkable that, on the elevated inland plains, the inflammation of some organ, we shall say the liver for instance, should only be attended by a continued inflammatory fever, while on the coast, during a season of aguish epidemics, it is mostly sure to be accompanied with a fever which, though continuous at its onset, yet in its course, after depleting and evacuating means have been used, is disposed to assume, and generally does, the intermitting or remitting type. Hence, we may infer that, to produce an intermittent fever, something more than local inflammation is essentially necessary: for these do not stand to each other in the positive relation of cause and effect. On the table-lands, ague is never known to originate, though inflammatory ailments are there most common; and, on the coast, intermittents and remittents are very common, without presenting any evidence of a co-existing local inflammation, though on some occasions these affections are conjoined.

There scarcely ever appears an acute disease in Lima which the women, who are exceedingly dexterous in the use of the Jeringa, or syringe, do not attack by clysters; and these during the intermission of fever are often resorted to with very beneficial results.

Emetics are seldom used except when the stomach is supposed to be in a foul state; and as the larger intestines are more usually considered in fault than the stomach, the vulgar attempt to relieve these by a very liberal use of enemata.

This administration of clysters is to a certain degree countenanced by those practitioners of the capital who, in direct opposition to the method commonly approved and followed by native practitioners of the highest reputation, make little use of purgatives in fevers of whatever denomination which happen to be attended with considerable excitement and vascular action; but who rather trust all to oily and emollient applications to the belly, with diluents, injections, warm baths, and the lancet.

After eight or ten days illness, should the patient, under this mode of treatment, begin to get better, and should the bowels come to unload themselves freely and without the help of purgatives, these gentlemen say, and say truly, "el enfermo evacua porque sana, y no sana porque evacua," that is, the patient's relief in his bowels is the consequence, and not the cause of his recovery.

But it is nevertheless true, that if such practitioners could only be prevailed upon to throw off their speculative notions derived from books and not from practice, as to the irritating effects of purgatives in cases of fever in general, and to release the bowels in the commencement of the attack, from the load of unhealthy deposit with which they are very usually encumbered in cases of intense fever, their patients would not fail to rejoice at the advantages gained by this practice. It will be found in a large proportion of cases, where the skin is dry and hot, and the fever of a continued or lightly remittent type, that nothing tends more than the discreet and early use of opening medicine, to relax the skin, and enable the existing fever, without any unnecessary procrastination, or waste of blood, to assume a milder and intermitting type.

Purgatives, as well as bleeding, are generally indispensable for the proper treatment of those forms of remittent and intermittent fever which are vulgarly called "*Tercianas en la Cabeza*," or tertian in the head. In these affections, delirium or great drowsiness, and bilious vomiting attend the exacerbation; and in some cases, particularly where the patient is of a corpulent habit, the disease is ushered in with bilious vomiting and a comatose state, which (until a doctor arrive to give assistance,) the women attack by common purgative clysters and cooling drinks. By such means, they often succeed in aiding nature to bring about a free sweat, which either terminates the paroxysm, or affords the patient a longer or shorter remission. But each successive paroxysm which returns with delirium or comatose symptoms, endangers the brain more and more; and when by the prompt administration of purgative clysters, (emphatically called "Ayudas," or helps,) and blood-letting, either general or local, the perilous determination of blood to the head is counteracted, and after this, vomiting stopped by iced refrigerants, according to the vulgar method, the first quiet interval should be used to give some suitable purgative. And thus it will be found that those disorders with well-marked cephalic symptoms, are commonly reduced to a very manageable state, and yield readily under the ordinary remedies; bark, in substance or infusion, and quinine.

The watery infusion of the Peruvian bark, called by the natives "*la tintura de la quina*," is in general a most suitable remedy during the intervals or remissions of intermittent and remittent fever; and when the stomach is irritable, this infusion is far more eligible than sulphate of quinine, which is sometimes, and particularly when long-continued, found to irritate the stomach very much. It is a prevailing opinion among native practitioners, that relapses are more apt to occur after an attack of *Terciana* has been removed by the sulphate of quinine, than when it has been stopped by the native bark, whether taken in powder or infusion.

When quinine is ordered, (after the bowels have been sufficiently attended to,) cooling lemonades at the temperature of the air are drank after each dose; or aperient beverages, such as cream of tartar in tamarind water, made agreeable with sugar, are preferred. Many of the natives are very partial to cold water and sugar, which they use by dipping a piece of sugar in the water, and slowly sucking or sipping at it till they have taken what they consider sufficient allowance. This they consider very refreshing, and have recourse to it in most cases of fever, whether they use quinine or not.

When quinine is given in form of pills, it may be worth while to recollect that these are often observed to pass through the bowels in an entire state, and, therefore, fail to effect the purpose for which they were given.

In many cases in which there are no distinct intermissions, an infusion of bark with the addition of a mild neutral salt, as sulphate of magnesia, answers every useful purpose; and in those instances where the intervals are complete, this combination of the bark and evacuant saves time, and is very appropriate.

It occasionally happens that after frequent relapses of a regular quotidian or tertian, the abdominal viscera become unusually irritated; the spleen or liver, and sometimes both, appear enlarged; and the fever, which now changes its type, becomes a severe remittent, or perhaps a stubborn quartan.

Where the disease from a more simple, thus took on a severer form—for example, when an intermittent had terminated in a remittent, I have witnessed the intestines in a torpid state, and at the same time puffy and inflated, whilst the head was much affected, and the mental faculties disturbed during the increase of the fever. In this state the patient's strength sunk, and the flesh rapidly fell away under the wasting force of a burning fever relieved by no cooling sweats. But mild mercurial inunction applied to the belly, soon affected very mildly the gums and salivary organs; and then all bad symptoms ceased. The secretions were improved, and perspiration was copious, though by all the variety of means previously resorted to, according to the particular views of different medical men, the cutaneous vessels could not be sufficiently relaxed. After this, no more remained to be done than to carry off the offensive contents of the bowels by the mildest laxatives,—manna dissolved in whey, repeated once or twice in the twenty-four hours, and thus a perfect recovery was obtained.

I have also found mercurial inunction prove most efficacious in ague complicated with hepatic obstruction, ascites, and great dilatation of the intestines; and of the treatment of ague and dysentery conjoined, I shall have occasion to take notice hereafter.

The above practical remarks are applicable to those fevers usually designated under the vulgar name of *Tercianas*. But yet, as such are known to appear under many varieties of character, to be removed by a diversity of remedies, and in some cases to produce much resistance to all ordinary methods of treatment, I shall offer a few observations to show what are the chief deviations from the most usual and regular appearance of those fevers of intermittent or remittent type.

When the fever is slight and without any well-marked gradations or stages, but returning at noted intervals, it is called *terciana boba*, or silly tertian. This variety sometimes presents itself with evening or nocturnal fever, accompanied with a good deal of headach. It is cured like ordinary and regular intermittents.

There is a sort of periodical *hemicrania*, which is of an intermittent nature, observing quotidian or tertian periods; and it yields to open bowels and bark, or Fowler's arsenical solution.

On some occasions the *terciana* is said to be seated in the stomach, *terciana en el estomago*. This particular appellation is given to those cases, where, during the exacerbations, or throughout the whole course of the fever, the gastric symptoms are the most prominent and urgent. Such cases may require, when the stomach is the seat of acute inflammation, to be in the first instance treated like *gastritis*; but, as in almost every case of this nature, the attack will be found to come on with the bowels more or less loaded, it will be proper from the beginning to follow the usual native practice of clearing out the intestines by moderately purgative clysters, and when the state of the stomach admits of it, a mild purgative may be given with the best results. Should the gastric symptoms subside greatly, during the intermission of a quotidian or tertian, the disorder may frequently be removed safely and readily by the infusion of bark with the sulphate of magnesia, and cooling drinks, such as lemonade, pine-ade, and tamarind-water, &c.

*Terciana en el ojo*, or tertian in one eye, is very particularly distinguished, and its presence is announced when an eye is the chief seat of uneasiness during the febrile and and periodical paroxysm. In such cases, all around about the eye appears livid after a tertian fit is over, or the eye may only look red and turgid.

This affection is sometimes treated as merely a local disease, and consequently becomes protracted and distressing, and by bleeding and purging, the patient is in such cases unnecessarily reduced. By giving quinine or bark during the intermissions, and attending to the bowels, this variety of terciana is readily removed.

Children at the breast do not always escape very troublesome agues, which are generally cured by dieting the nurse. Tincture of quinine is frequently, and sometimes with good effect, applied externally to the child by gentle friction.

In orchards round town, older children who are capable of helping themselves to fruit, and allowed to play in the humid or shaded parts of these enclosures, suffer greatly from agues. Among these little patients more particularly, though like cases may also be seen in the city, are some quite jaundiced, and others are seen with ague-cakes very painful, at times, to the touch, during the heat of a paroxysm. In those affected in the latter manner, the vomiting is frequently very severe, beginning in the cold, and continuing through the hot stage, and the type itself is irregular and changeable; at one time quartan, and at other times tertian or quotidian.

I have been called to see a child who, for four days, had laboured under a double quotidian, leaving a distinct remission. By means of a laxative clyster to begin with, followed by an emetic, which was succeeded by a mild opening medicine and mallow decoction for drink; and by means of the warm bath when the fever was highest, or skin hottest, the fever assumed the type of a regular quotidian ague: each exacerbation ending in a general sweat. But, at first, until the bowels were cleared out and regulated, the general state of the skin was dry, the patient having only slight partial sweats after each return of exacerbated fever, attended with burning forehead, headach, and drowsiness. The case, once changed in type, easily yielded to quinine.

In Lima, I was called to attend a young man from Chili, who was attacked by semitertian in consequence of gigers having entered his toes; the local irritation excited by these insects having induced sympathetic glandular swellings in the groin, with fever terminating in semitertian. Every day there was an exacerbation, but only every other day a perfectly formed paroxysm, consisting of cold, hot, and sweating stage. The fever continued after the gigers were extracted or destroyed, the wounds occasioned by them healed, and the sympathetic swelling resolved; but in two or three days it was removed by the common bark infusion, with the addition of a neutral salt, which served to keep the bowels open. And it may be noticed by the way, that it is rarely requisite, with a view to restrain excessive alvine dejections, to conjoin opiates with the infusion of bark in the *tercianas* of Lima.

I have known a stout woman, of mixed Indian and European blood, who never had an attack of ague unless it was occasioned by anger; and having first commenced on the coast, it afterwards came on indifferently, whether on the coast or in the elevated villages of the interior. A fit of choleric or angry passion always brought on a relapse, which she was accustomed to remove by ordinary clysters, followed by the bark infusion with Epsom salts.

I have known an Indian farmer, of herculean strength, who, when attacked by *tercianas*, derived no benefit from the regular prescriptions of the faculty, but was always cured by ordinary clysters and a dram of aguardiente or pale brandy. Many are cured of obstinate agues by the juice of sour oranges. It is firmly believed by the natives, that relapses are sure to be caused by drinking milk; and therefore no quantity of milk, however small, is allowed in tea or coffee taken by the aguish patient.

Some persons affected with *tercianas* are always injured by bark and cured by ices or cooling acidulated drinks; but these individuals take good care to clear the bowels with clysters, or perhaps a mild laxative, before ices are resorted to, or considered safe. Others there are, who, after their bowels have been acted upon by clysters, are accustomed to take an emetic, and then they trust the rest to diet and *frescos*, or cooling beverages.

I have known a beef-steak dinner, with a good allowance of port wine after it, cure a chronic ague. But this is a remedy that often does more harm than good; for when it does not cure quickly, it seldom cures at all, and may increase those visceral disorders which are frequently attendant on obstinate quartans, in which more good may be expected from blue pill than generous living.

It is related of a physician who long practised in Lima, and was subject to a slight *terciana* for twenty years, that when it left him he only survived a few days.

I have conversed with a respectable ecclesiastic of the same city, who, after having long suffered from a tertian that baffled the skill of the faculty, was at length cured by applying some sweet-smelling flowers to his nostrils, as soon as he felt the usual precursory uneasiness at stomach and chillness disconcert his frame. The writer stopped this disorder in his own case by employing the vapour-bath in the cold stage, and so forcing a sweat. Not a few have been cured of long continued ague by bathing in the sea during the cold fit.

Obstinate intermittents contracted about the period of the autumnal or vernal equinoxes, are many times cured by sea-bathing, after all other remedies have failed. Sometimes a voyage to Chili is undertaken with a view to fortify the constitution, and insure a permanent recovery. A journey to the interior of Peru is not found to answer the same purpose; and in the frigid mining districts, the bilious vomiting is very severe during an attack of ague. I once, when long out of health, with a frequently recurring quotidian, undertook a journey to the interior and temperate valleys, but did not find that this change of climate rendered the fever less apt to return, though it increased the general tone of the system. But I experienced, that while it had the effect of strengthening the cold stage, the bilious vomiting and gastric disturbance were much less in these temperate situations than on the coast, where the cold fit is sometimes but faintly marked, while the hot fit is long and distressing. In Lima, it is understood that delicate persons, and such as are subject to relapses of *tercianas*, should take refuge during the winter in the neighbouring village of Chorrillos.* Such, indeed, is the general liability or propensity to *tercianas* in Lima, and especially in that part of it called the Cercado, at certain seasons, that when these prevailed epidemically, I have observed a fever which preceded a general eruption of urticaria, assume the intermittent type. And patients debilitated by other diseases, while going about as convalescents, are very often seized, particularly if they commit any irregularity in diet, with some form of intermittent. But, to conclude these observations, relapses of ague are so common, from the slightest causes of change in the warmth of the surface, however induced, whether arising from changes in dress, or different states of the atmosphere, that it appears nothing is more essential to complete convalescence, or necessary to guard against relapses of the disease, than keeping up an equable warmth of the feet and surface of the body.—*Edinburgh Med. and Surg. Journal.*

---

*On a remarkable Diminution of the Medulla Oblongata, and adjacent portion of the Spinal Marrow, consequent upon Spontaneous Dislocation of the Processus Dentatus, and Ankylosis at the upper part of the Spine—yet unattended with any symptom of Paralysis.* By P. D. Handyside, M. D.—William Cragie, a working-cutler by trade, suffered, at the age of 22, under a protracted attack of rheumatic fever, and was in consequence confined to bed for about seven weeks. During much of that time he was so debilitated as to be unable to turn his head or body, and lay almost constantly on his left side. On recovering from this illness, there was evident ankylosis between the occiput, atlas, and adjoining vertebræ, his head being bent immoveably forwards, and slightly to the right side. Nevertheless, he was able soon to return to his accustomed employment, and, indeed, he performed equally well the more severe and laborious operations at his forge, and the nicer and more delicate manipulations of a surgical instrument-maker, at which department of his trade he was unusually dexterous.

Four years previous to his death, he suffered under a severe bronchitic attack. After repeated invasions of this complaint, to which the severity of his occupation, with the alternations of temperature which he was frequently called upon to endure, in a great degree exposed him, he was at length carried off, after a day of continued bodily fatigue, by a very sudden and rappid attack of asphyxia. Thus, at the age of 32, terminated a case of what was found, upon dissection, to be advanced vesicular bronchitis.

*Sectio.*—Upon examining the upper part of the spine, the occipital bone, as was anticipated, is found firmly ankylosed to the atlas. These two bones are immoveably united in three places. The ankylosis serves to connect the condyloid processes, and the posterior fourth of the foramen magnum of the occipital bone, with the corresponding surfaces of the atlas; and the circumstance of the head of this individual being retained immoveably in a direction downwards and forwards to the right side, is explained by the circumstance of the right inferior articular surface of the atlas being dislocated downwards and forwards over the corresponding articular surface of the axis, and retained in this false position by a strong bony union.

The displacement between the atlas and axis is such, that the anterior portion of the ring of the former bone projects forwards, and to the right side, from the corresponding portion of the body of the latter, to the extent of nine lines. Again, the space for the lodgment of the lower part of the medulla oblongata, which intervenes between the posterior surface of the odontoid process of the axis, and the anterior surface of the posterior segment of the atlas is reduced, in the mesian plane, to two lines; while to the right side of the mesian line, where, indeed, the medulla oblongata lay, it does not exceed three lines and two-thirds in its antero-posterior diameter.

The synovial membrane of the atlanto-odontoid articulation is wanting, owing to the separation of these surfaces and their relative position being so much altered and deranged. However, the intervening space which has resulted from this distortion is occupied by a very strong fibro-cartilaginous band, of a cuboid shape, six lines in length by five lines in

---

*See Chorrillos, described in "Peru as it is," vol. i. p. 90.

breadth, presenting in its entire length, and imbedded in its dense texture, four vertical laminæ of bone, at their extremities connected to, and serving to retain immoveably, what were formerly the two opposite articular surfaces. The transverse ligament of the atlas is altogether wanting, but the tubercles which served for the attachment of that ligament are connected by soft fibro-cellular tissue to the intervening fibro-cartilaginous band already noticed. The ligamentum subflavum, extending between the atlas and axis, is absent, except in the mesian line, where an elongated narrow band, remarkably dense, firm, and resisting in texture, though retaining none of its original elasticity, is stretched between these two bones posteriorly, in a situation where they are apart from each other to the distance of one inch and three-eighths.

The articulations intervening between the second, third, fourth, and fifth vertebræ of the neck, were not very free in their movements, owing to the deposition of calcareous matter in the fibro-cartilages covering their surfaces. An approximation to ankylosis is sufficiently manifested in this condition of the parts.

The membranes and vessels of the medulla oblongata and cervical portion of the spinal marrow were natural.

The medulla oblongata itself appeared natural, with the exception of a considerable alteration in the antero-posterior diameter of its lower portion, which, from the unusually contracted limits of the spinal canal at its upper part, was reduced to only three lines in thickness. It was also remarkably altered in its position, for it rested obliquely on the basilar portion of the occipital bone, while its lower portion was placed altogether to the right side of the odontoid process of the axis and of the arch of the atlas in a circumscribed space, the antero-posterior diameter of which, as already remarked, did not exceed three lines and two-thirds, and the transverse diameter of which measured only six lines.

The nerves arising from the medulla oblongata were of their natural size, consistence, and colour, and so likewise were the cervical nerves.

No paralysis, or even any remote indication of the existence of that state, or of any other affection of the nervous system, presented itself in any part of the body.

The foregoing case serves to illustrate well the very considerable amount of pressure and torsion which the medulla oblongata and upper part of the spinal marrow may be brought gradually to sustain, without the production of the slightest inconvenience; although these important and essential parts of the nervous system, being subjected suddenly to even a moderate amount of pressure, produces an interruption, which, if maintained, may terminate speedily in a total cessation of the vital functions of the body.

A case, allied somewhat to that under consideration, in the phenomena which it exhibited during the life of the patient, is detailed by Mr. Phillips of London,* and is referred to in this Journal, (vol. xlix. p. 265,) in which there existed a fracture and great displacement, not only of the *atlas* but of the *processus dentatus* likewise. Although the relations of this process to the displaced portions of the atlas and axis are not described in the account referred to, yet it is stated that there was no diminution in the width of that part of the spinal canal which lodged the medulla oblongata, but, on the contrary, this space was, in the region of the atlas, at the mesial line, actually more capacious than natural. "There was no lesion of the spinal chord found at death,"—a fact which serves in some degree to explain how, during life, no lesion of sensation or motion presented itself, and how there was no material disturbance of the functions of the economy.

Caries of the atlas and axis, accompanied with spontaneous dislocation of the processus dentatus, among other pathognomonic signs, is characterised by the entire absence of paralysis, which, according to Professor Syme, is a very constant mark of the existence of this very interesting and important disease. The case which I have here detailed is worthy of notice, as presenting the peculiarity just noticed, while, at the same time, the history, symptoms, and necroscopic appearances of caries fail to be traced in it.

In the museum at Guy's Hospital, London, are some preparations illustrative of ankylosis and exostosis of the cervical vertebræ. But in all these cases paralysis had resulted. Dr. Hodgkin, the author of the catalogue of that valuable collection, describes the preparations marked Nos. 1011 and 1012, as exhibiting the processus dentatus so much enlarged as to have occasioned paralysis. In No. 1012, a preparation taken from the body of a patient who had been under the care of Dr. Bright, partial paralysis, both of the upper and lower extremities, had been produced by an exostosis arising from the processus dentatus.—*Edinburgh Med. and Surg. Journal.*

---

*A Professional Accumulation.*—The celebrated Doctor Grate, who lately died at Hanover, left the enormous fortune of three millions six hundred thousand Prussian dollars,—equal to thirteen millions of francs, or more than half a million sterling,—which he amassed almost entirely from his professional earnings, having began life with a fortune of not more than eight or nine thousand pounds sterling.

---

*London Medico-Chirurg. Trans. vol. xx. p. 78.

